# Rare genetic variants in *PKD1* and *SMAD2* are associated with intracranial aneurysms in the general population

**DOI:** 10.1177/17474930251334501

**Published:** 2025-04-02

**Authors:** Bibi M Wolters, Mark K Bakker, Kristiina Rannikmäe, Paul J Hop, Ynte M Ruigrok

**Affiliations:** 1 Department of Neurology and Neurosurgery, University Medical Center Utrecht Brain Center, Utrecht University, Utrecht, The Netherlands; 2 Centre for Medical Informatics, Usher Institute, The University of Edinburgh, Edinburgh, UK

**Keywords:** Subarachnoid hemorrhage, genetics, intracranial aneurysm, bioinformatics, stroke, prevention

## Abstract

**Introduction:**

Family studies identified several rare genetic risk variants for intracranial aneurysms (IAs) and aneurysmal subarachnoid hemorrhage (ASAH). In addition, certain monogenic disorders caused by rare penetrant genetic variants predispose individuals to IA and ASAH. We investigated the effect of these variants on IA and ASAH in the general population.

**Patients and Methods:**

We tested the association between genetic variants within IA-associated genes and IA and ASAH using a burden test, sequence kernel association test (SKAT), and variant-level aggregated Cauchy association test (ACAT-V) in the UK Biobank. Variants were stratified by allele frequency and predicted impact on the protein structure. Sensitivity analyses were performed on only ASAH patients and excluding participants diagnosed with an aforementioned monogenic disorder.

**Results:**

In the group of 1656 IA cases, including 928 ASAH cases, and 391,948 controls, associations were identified for ultrarare variants with moderate or high impact in *PKD1* (odds ratio (OR) = 1.42; 95% confidence interval (95% CI)= 1.06–1.85, p = 4.28 × 10^−7^ (SKAT)) and *SMAD2* (OR = 4.89; 95% CI = 1.63–11.05, p = 7.10 × 10^−5^ (SKAT)). Upon excluding participants diagnosed with the respective monogenic disorders, these associations remained. When considering only ASAH cases, the association with *SMAD2* was similar (OR = 4.85; 95% CI = 1.02–13.7; p = 9.0 × 10^−4^) while for *PKD1* the association diminished (OR = 1.29; 95% CI = 0.85–1.87; p = 0.043).

**Discussion and Conclusion:**

Ultrarare damaging variants in *PKD1*, a gene causing autosomal dominant polycystic kidney disease, and *SMAD2*, a gene causing Loeys-Dietz syndrome, were associated with IA in the general population, even in the absence of a diagnosis of these disorders. Our results may contribute to the development of genetic screening methods for IA in a clinical setting.

## Graphical abstract

**Graphical abstract:**
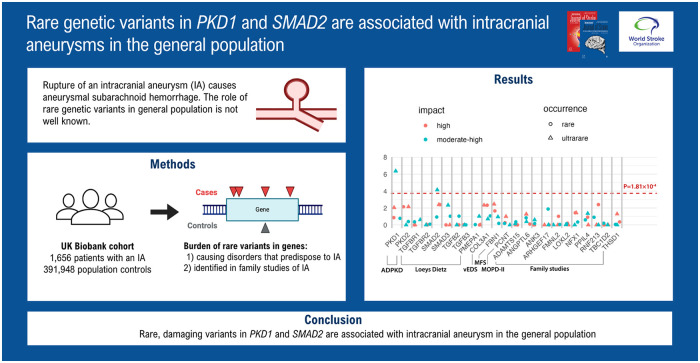

## Introduction

Rupture of an intracranial aneurysm (IA) causes aneurysmal subarachnoid hemorrhage (ASAH), a severe type of stroke. Due to its occurrence in relatively young people, with a median age of onset at 50 years,^1^ and a high case fatality rate of 31%,^2^ ASAH significantly impacts the number of productive life years, despite accounting for only 5% of all stroke cases.^1–3^ ASAH can be prevented by early detection and treatment with surgical clipping or endovascular coiling of an unruptured IA (UIA). UIAs are relatively common, with a prevalence of approximately 3% in the general population.^3^

Genetic factors play a substantial role in the etiology of ASAH, as demonstrated by a twin-based heritability estimate of 41%.^4^ The heritability of ASAH is partly attributed by rare, penetrant mutations, with a large effect on the liability to UIA and/or ASAH.^1^ Several rare monogenic disorders exhibit an inherent predisposition for UIA and ASAH,^1^ suggesting a link between the genes implicated in these disorders and the manifestation of UIA and ASAH. These disorders and their causal genes include autosomal dominant polycystic kidney disease (ADPKD) genes *PKD1* and *PKD2*; Loeys-Dietz syndrome (LDS) genes *TGFBR1*, *TGFBR2*, *SMAD2*, *SMAD3*, *TGFB2*, *TGFB3*, and *PMEPA1*; vascular Ehlers–Danlos syndrome gene *COL3A1*; Marfan syndrome gene *FBN1*; and Microcephalic/Majewski’s Osteodysplastic Primordial Dwarfism Type II gene *PCNT*.^1,5^ It is unknown whether genetic variants in the genes causing these disorders cause UIA and ASAH independent of a diagnosis of the respective disorders.

In addition, analysis of rare genetic variants segregated within families with a high burden of IA has identified several variants in genes such as *ADAMTS15, ANGPTL6*, *ARHGEF17*, *ANK3*, *FMNL2*, *LOXL2*, *NFX1*, *PPIL4, RNF213, TBC1D2*, and *THSD1*.^1,6–8^ It remains unclear to what extent these genes contribute to UIA and ASAH in the general population and whether they can be utilized to identify high-risk patients.

To answer the questions about the contribution of these genes in IA, we performed a gene-level rare-variant burden analysis using data from the UK Biobank.^9^ We tested the association between variants in genes linked to UIA and ASAH in both monogenic disorders and family studies, and UIA and ASAH in the general population.

## Methods

### Methods overview

**Figure 1.**
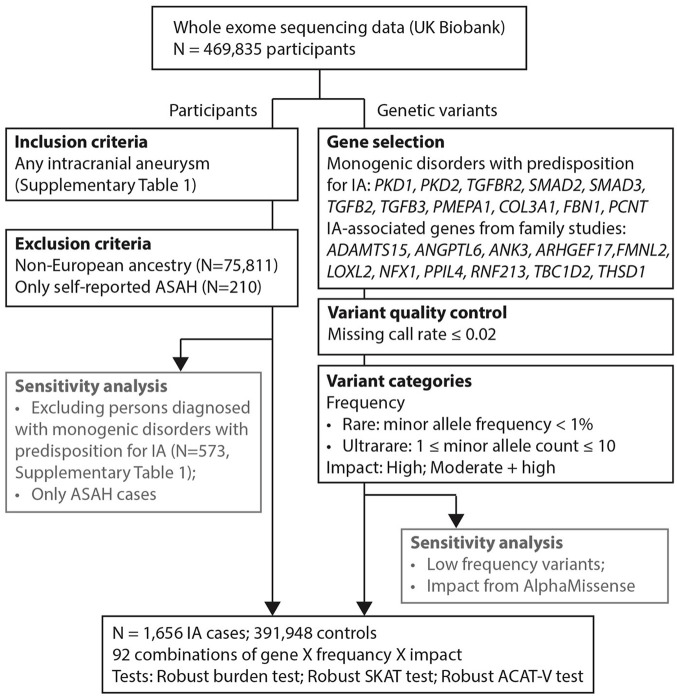
Flow chart of the methodology. ASAH: aneurysmal subarachnoid hemorrhage. Inclusion and exclusion criteria led to a study cohort of 393,604 individuals, including 1656 intracranial aneurysm (IA) cases. Variant quality control was performed before grouping variants based on allele frequency and impact. A Burden test, Sequence Kernel Association Test (SKAT) test, and aggregated Cauchy association test variant-level (ACAT-V) test were performed for every group.

The manuscript was written in accordance with the STREGA (STrengthening the REporting of Genetic Association Studies) guidelines for reporting genetic association studies.^10^ Figure 1 provides a graphical representation of the steps involved in the methodology. Below, we provide details on the study population, variant selection and annotation, and main statistical analyses. Additional details on single-variant analysis and description of sensitivity analyses can be found in the Supplementary Data.

### Study population

The study population consisted of participants included in the UK Biobank, a prospective cohort study comprising approximately 500,000 participants of ages 40–69 at the start of follow-up, from whom whole exome sequencing (WES) data was obtained.^9^ We identified all individuals diagnosed with an IA, either ruptured or unruptured, using the International Classification of Diseases (ICD)-10 codes I67.1 (cerebral aneurysm, unruptured) and I60 (subarachnoid hemorrhage). We restricted our analysis to participants of European ancestry (Supplemental Table 1). Since we identified patients by hospital codes and not all participants received brain imaging, persons with undetected, unruptured, IAs may be present among the controls. In addition to this main analysis, we evaluated whether any gene for which we found an association with IA was also associated with IA when we excluded patients with a diagnosis of any of the monogenic disorders that predispose to IA.

### Selection of genes to investigate

We used whole exome sequencing data provided by the UK Biobank (the 500K exome release) as genetic input data to assess the following gene sets: (1) genes involved in monogenic disorders that exhibit an inherent predisposition for IA: ADPKD genes *PKD1* and *PKD2*; LDS genes *TGFBR1*, *TGFBR2*, *SMAD2*, *SMAD3*, *TGFB2*, *TGFB3*, and *PMEPA1*; vascular Ehlers-Danlos syndrome gene *COL3A1*; Marfan syndrome gene *FBN1*; and Microcephalic/Majewski’s Osteodysplastic Primordial Dwarfism Type II gene *PCNT*^1,5^ and (2) genes that segregate with IA or ASAH in families: *ADAMTS15, ANGPTL6*, *ARHGEF17*, *ANK3*, *FMNL2*, *LOXL2*, *NFX1*, *PPIL4, RNF213, TBC1D2*, and *THSD1*.^1,6–8^

### Variant annotation and selection

The SnpEff tool was employed for variant annotation and effect prediction of genetic variants within our selected genes.^11^ Whole exome sequencing genotype calls provided by the UK Biobank were obtained and have been described before.^12^ Prior to variant annotation, variants with missing call rates exceeding 0.02, variants with minor allele frequency (MAF) above 0.01, and monomorphic variants were removed. Annotation of the VCF files was conducted by SnpEff utilizing the pre-built database GRCh38 version 105 and based solely on canonical transcripts. We differentiated between rare variants, characterized as single nucleotide variants (SNVs) with an MAF below 0.01, and ultrarare variants, identified as SNVs with a minor allele count (MAC) of up to 10. These categories cover the allele frequency spectrum not captured by common variant association studies of IA.^1^ Two impact categories were defined: (1) high impact, meaning protein-coding variants with a predicted impact of “HIGH,” excluding structural interaction variants, and (2) high impact or moderate impact, meaning protein-coding variants with a SnpEff-predicted impact of “HIGH” or “MODERATE.” As a sensitivity analysis, we performed a test using variant effect predictors from AlphaMissense (see Supplementary Data for the methods).^13^

### Main statistical analysis

We employed gene-based tests that collectively analyze all selected variants within a gene using three distinct gene-level tests: a burden test using Firth logistic regression, Sequence Kernel Association Test (SKAT) test, and aggregated Cauchy association test variant-level (ACAT-V).^14,15^ All analyses used IA (i.e. UIA and ASAH grouped together) as the primary outcome, and the genic variant burden as the independent variable. A detailed description of the tests can be found in the Supplementary Data. The tests are implemented in the R package “rare variant analysis toolkit” (RVAT).^16^

Genetic principal components 1–10, provided by the UK Biobank, and sex were used as covariates. The combination of three statistical tests, two variant frequency bins, two impact bins, and 23 genes resulted in 276 performed tests, established a Bonferroni-corrected p-value threshold for statistical significance of 1.81 × 10^−4^. Since each of the three statistical approaches utilized an aggregation-based score metric, no odds ratio (OR) is obtained. Therefore, we also performed a Firth logistic regression with the purpose of calculating the OR of variant burden on IA liability—representing the change in probability of having an IA for each additional carried variant. The number of variant carriers in cases and controls was calculated to determine the true and false detection rate and positive predictive value (true positives/variant carriers).

## Results

### Number of participants and variants

Following quality control and variant categorization, 1656 IA patients (721 UIA and 925 ASAH) and 391,948 controls were identified. The baseline characteristics of the study cohort are listed in Table 1. The total variant count per test group (combination of gene, rarity, and impact) ranged from 5 to 3672 (mean = 372; SD = 683) and the total number of variant carriers per group exhibited a range of 6 to 110,090 (mean = 5.99 × 10^3^; SD = 1.66 × 10^4^). The number of variants and variant carriers in cases and controls are shown in Supplemental Table 2.

**Table 1.** Basic demographics and characteristics of individuals in the study cohort.

	Main analysis cohort		Excluding participants with monogenic disorders	
	IA cases	Controls	IA cases	Controls
Individuals (n)	1656	391,948	1646	391,385
ASAH (n, %)	928 (56%)	0 (0%)	925 (56%)	0 (0%)
Men (n, %)	612 (37%)	180,323 (46%)	608 (37%)	180,054 (46%)
Age (median; Q1, Q3)	74.7 (67.8, 79.0)	72.8 (65.1, 77.8)	74.7 (67.9, 79.0)	72.8 (65.1, 77.8)
Family history of stroke (n, %)	514 (31%)	107,824 (28%)	511 (31%)	107,655 (28%)
Diastolic blood pressure (mean, SD)	82.8 (10.4)	82.3 (10.1)	82.8 (10.4)	82.3 (10.1)
Systolic blood pressure (mean, SD)	140.4 (18.4)	138.3 (18.6)	140.4 (18.4)	138.3 (18.6)
Pack years adult smoking as proportion of life span (mean, SD)	0.30 (0.45)	0.17 (0.34)	0.30 (0.45)	0.17 (0.34)

The two rightmost columns show the demographics of the study population excluding patients with polycystic kidney disease, Ehlers–Danlos syndrome, Marfan’s syndrome, or other specific congenital malformation syndromes including Loeys–Dietz syndrome.

SD: standard deviation; IA: intracranial aneurysm; ASAH: aneurysmal subarachnoid hemorrhage; Q1, Q3: quartile 1, quartile 3.

### Association between IA and putative IA risk genes

**Figure 2.**
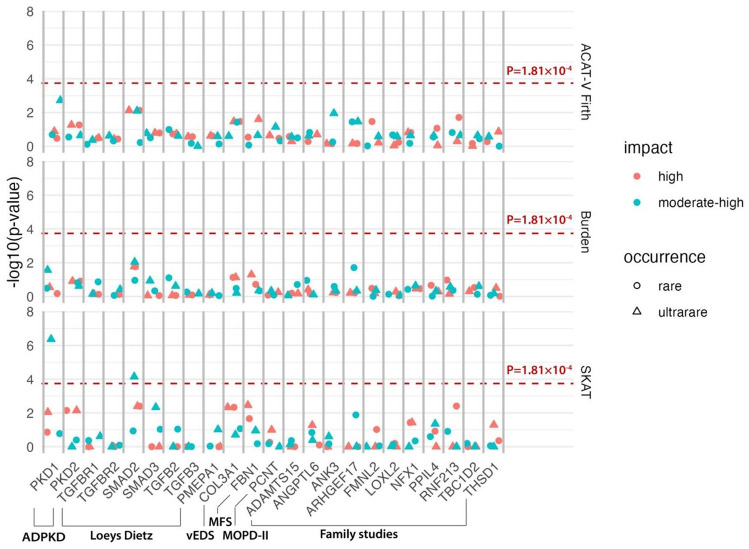
Association between putative risk genes and intracranial aneurysms. ADPKD: autosomal dominant kidney disease; vEDS: vascular Ehlers–Danlos syndrome; MFS: Marfan syndrome; MOPD-II: Microcephalic/Majewski’s Osteodysplastic Primordial Dwarfism Type II. Gene-based associations with intracranial aneurysms (IAs) were tested. The vertical axes indicate −log10(p-value) of the associations. Results for three statistical tests are shown: ACAT-V Firth (top), Burden (middle), and SKAT (bottom). Colors indicate the impact category, and shape indicates the variant frequency category. The dashed red line indicates the Bonferroni-corrected p-value threshold of 1.81 × 10^−4^.

Statistically significant associations between ultrarare variants with moderate or high impact in the *PKD1* and *SMAD2* genes and IA were found using the SKAT test (Figure 2 and Supplemental Table 3) as further detailed in the next two sections. On using the Burden test and the ACAT-V test, no statistically significant associations between any of the genes and IA were found. Also, when analyzing rare variants, no statistically significant associations were found.

#### PKD1

**Figure 3.**
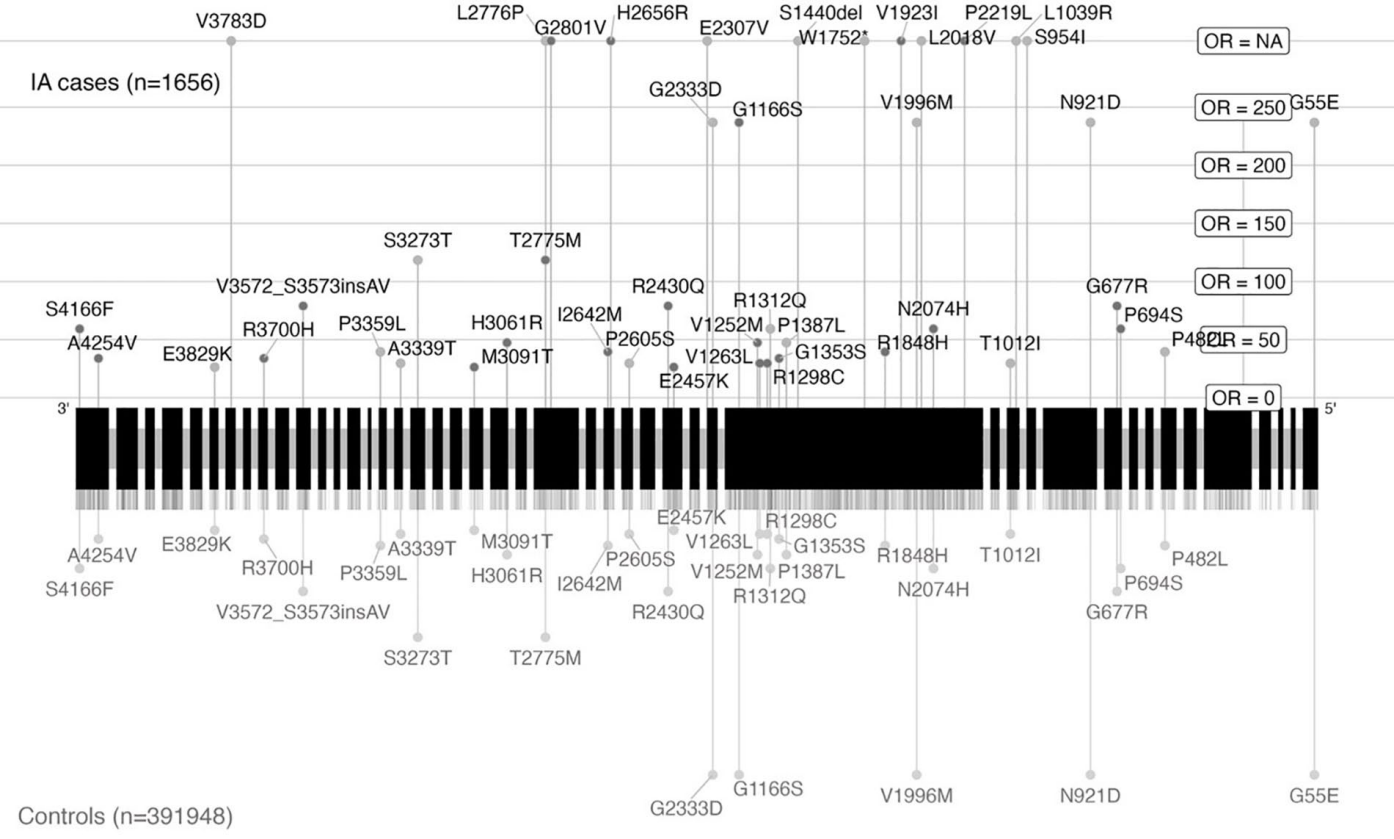
Genetic location of ultrarare variants with moderate or high impact on gene PKD1 visualized based on canonical transcript ENST00000262304. All exons are shown. Variants carried by intracranial aneurysm (IA) cases are represented by lines above the exon blocks with the length depicting the variant-level odds ratio (OR). Dark points highlight the variants carried by at least one aneurysmal subarachnoid hemorrhage (ASAH) case. Variant labels indicate the amino-acid substitutions with a Human Genome Variation Society (HGVS) notation. Lines representing variants carried by both cases and controls are mirrored below the exon blocks. Variants carried solely by controls are indicated by short ticks.

In the main study cohort, we found 46 IA cases and 7427 controls carrying at least one 44 distinct ultrarare moderate- or high-impact variant in the *PKD1* gene (OR = 1.42 (95% CI = 1.06–1.85; p = 4.28 × 10^−7^ (SKAT)); Figure 3 and Table 2). These variants covered most of the *PKD1* gene. The single-variant association analysis indicated that the observed gene-wide association was not driven by one or a small number of variants (Supplemental Table 4). When considering only high-impact variants, nominally statistically significant (p < 0.05) associations were found using the ACAT-V and burden tests. On analyzing ASAH cases only (*N* = 23 cases with a *PKD1* variant), a comparable association was found (OR = 1.29, 95% CI = 0.85–1.87, p = 4.35 × 10^−2^ (SKAT); Table 3 and Supplemental Table 3), although no longer statistically significant. Our sensitivity analysis showed that the association did not remain when considering variants with a likely pathogenic effect based on AlphaMissense (N = 3 cases carriers, 667 control carriers, p = 0.66, Supplemental Table 5). When considering only participants without a diagnosis of monogenic disorders predisposing to IA, the association remained statistically significant (OR = 1.36, 95% CI = 1.00–1.79, p = 1.33 × 10^−5^; Supplemental Table 6). The sensitivity analysis including low-frequency variants showed that the observed association was limited to ultrarare variants (Supplemental Table 7). Only a nominally significant (p < 0.05) association was found with another ADPKD-related gene, *PKD2* (Supplemental Table 3).

**Table 2.** Characteristics of the datasets used to test the association between intracranial aneurysms, and the genes PKD1 and SMAD2.

Association test	IA—ultrarare-, moderate-, or high-impact variants in PKD1			IA—ultrarare-, moderate-, or high-impact variants in SMAD2		
	Total	Cases	Controls	Total	Cases	Controls
Cohort	393,604	1656	391,948	393,604	1656	391,948
Variants	2788	44	2776	122	4	122
Variant carriers	7473 (1.90%)	46 (2.78%)	7427 (1.89%)	225 (0.06%)	4 (0.24%)	221 (0.06%)
N carrying > 1 variant	198 (0.05%)	0	198 (0.05%)	0	0	0
Variants carried per variant carrier	1–3	1	1–3	1	1	1

IA: intracranial aneurysm; N: sample size.

**Table 3.** Characteristics of the datasets used to test the association between aneurysmal subarachnoid hemorrhage, and the genes PKD1 and SMAD2.

Association test	ASAH—ultrarare-, moderate-, or high-impact variants in PKD1			ASAH—ultrarare-, moderate-, or high-impact variants in SMAD2		
	Total	Cases	Controls	Total	Cases	Controls
Cohort	392,876	928	391,948	392,876	928	391,948
Variants	2780	23	2776	122	2	122
Variant carriers	7450	23	7427	223	2	221

ASAH: aneurysmal subarachnoid hemorrhage; N: sample size.

#### SMAD2

**Figure 4.**
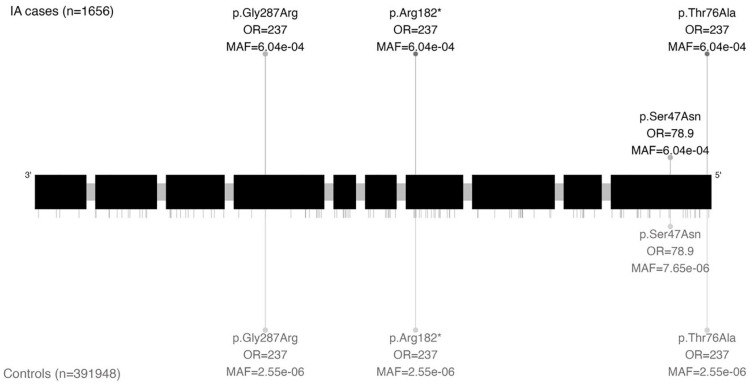
Genetic location of ultrarare variants with moderate or high impact on gene SMAD2 visualized based on canonical transcript ENST00000262160. All exons except exon 1 are shown. Variants carried by intracranial aneurysm (IA) cases are represented by lines above the exon blocks with the length depicting the variant-level odds ratio (OR). Dark points highlight the variants carried by at least one aneurysmal subarachnoid hemorrhage (ASAH) case. Variant labels indicate the amino-acid substitutions with a Human Genome Variation Society (HGVS) notation, the variant-level OR, and the minor allele frequency (MAF) in cases/controls. Lines representing variants carried by both cases and controls are mirrored below the exon blocks. Variants carried solely by controls are indicated by short ticks.

In the main study cohort, we found four IA cases and 221 controls who carried one of four distinct ultrarare variants with moderate or high impact in the *SMAD2* gene (OR = 4.89, 95% CI = 1.63–11.05, p = 7.10 × 10^−5^ (SKAT); Figure 4 and Table 2). The variants all had highly similar effect sizes and MAFs, showing no evidence that the signal was driven by a single variant (Supplemental Table 4). When considering only high-impact variants, nominally statistically significant (p < 0.05) associations were also found using all tests, regardless of the frequency of the variants. Our sensitivity analysis showed a consistent effect when considering likely pathogenic variants based on AlphaMissense (N = 2 case carriers, 95 control carriers, p = 0.002 (SKAT), Supplemental Table 5). The four variants carried by IA cases were localized in three distinct exons across the gene, rather than being concentrated within a specific section. Two of these variants were located on the MH1 domain (p.Ser47Asn, p.Thr76Ala), one on the MH2 domain (p.Gly287Arg), and one just downstream of the MH1 domain (p.Arg182*). On analyzing the ASAH cases only, a nominally statistically significant association was found with a similar effect size (OR = 4.85, 95% CI = 1.02–13.7, p = 8.99 × 10^−4^ (SKAT); Table 3 and Supplemental Table 3). When considering participants without a diagnosis of monogenic disorders predisposing to IA, the association remained (OR = 4.93, 95% CI = 1.64–11.1, p = 6.79 × 10^−5^; Supplemental Table 6). The sensitivity analysis including low-frequency variants in *SMAD2* showed no association with IA (Supplemental Table 7). Only a nominally significant association was found with two other genes linked to LDS (Supplemental Table 3).

## Discussion

We investigated whether rare genetic variants in genes previously linked to IA (i.e. both UIA and ASAH) were also associated with IA in the general population. We found associations between IA and rare, damaging variants in *PKD1*, one of the two disease-causing genes for ADPKD, and *SMAD2*, one of the disease-causing genes for LDS. Notably, these associations remained in the absence of a diagnosis of the monogenic diseases ADPKD or LDS. On analyzing ASAH patients only, the associations also remained, although for the *PKD1* gene, this association was no longer statistically significant. We did not find an association between IA and rare damaging variants in genes previously identified through family studies.

Mutations in the *PKD1* gene are known to cause ADPKD.^17^ *PKD1* encodes a polycystin protein involved in calcium channel regulation, intracellular calcium balance, and cell-cell/matrix interactions.^18^ Our findings suggest an association between *PKD1* and IAs, even in individuals without an ADPKD diagnosis. Future research should clarify if this results from (1) undiagnosed ADPKD due to misdiagnosis or mild/prodromal form or (2) separate mechanisms where *PKD1* mutations independently predispose to ADPKD and IA. The latter could occur if mutations in different regions of the *PKD1* gene differentially affect ADPKD and IA. Previous studies reported inconclusive evidence whether liability to IA depended on the location of *PKD1* mutations.^19,20^ We found no regional mutation specificity for *PKD1* variants in IA patients, but our study lacked the statistical power to test for regional enrichment. In addition, we found no association between IA and rare, damaging *PKD2* variants.

*SMAD2* is implicated in LDS, a rare autosomal dominant connective tissue disorder affecting the cardiovascular, ocular, and skeletal systems.^21^ LDS results from mutations in genes involved in the transforming growth factor beta (TGF-β) pathway.^22^ *SMAD2* is a transcription factor that contributes to TGF-β-mediated gene transcription. Previous research has shown that TGF-β mainly drives thoracic aneurysm formation in LDS,^21^ although aneurysms in other locations such as the intracranial arteries have been observed.^23^ No conclusive evidence of an increased IA risk has been established. Our findings indicate a direct association between rare, damaging *SMAD2* variants and the formation of IAs, implicating TGF-β signaling in IA formation. As with *PKD1*, future research should determine whether the observed association of *SMAD2* with IAs is due to missed diagnoses or prodromal effects of LDS, or whether certain mutations of the *SMAD2* gene predispose to IA independent of the typical LDS symptoms. Seven *SMAD2* variants have been previously reported as likely pathogenic for LDS, all located on the MH2 domain.^22,24,25^ None of these variants were identified among IA cases in our study. The observed association was driven by four different variants, only one of which was located within the MH2 domain (p. Gly287Arg).

We did not find an association between IA and variants in genes previously associated with IA in family studies. A study investigating index variants, but no additional variants, in these genes also found no evidence for their role in large Dutch and UK case series.^26^ Another study showed that six distinct variants in *ANGPTL6*, a gene previously associated with IA in a family study, were carried by 9 out of 265 (3.3%) IA cases with a positive family history.^27^ In our study, we did not find this association, which may be because we included all IA patients regardless of family history of IA. Another reason for the lack of associations between IA and genes identified in family studies may be that patients with familial IA tend to have an ASAH at a younger age (46.5 vs 50.8 years for familial and non-familial ASAH, respectively).^28^ With an inclusion age of 40–69 years in the UK Biobank, some patients with family history of ASAH may have deceased before reaching the point of inclusion, thereby depleting the group of older patients.

Our study had several limitations. First, SKAT results could be inflated by variants with extremely large effect sizes, such as ultrarare variants. A significant SKAT result but not ACAT-V or burden result could occur if variants with opposing effect sizes are present. However, our single-variant analysis did not identify rare damaging variants with a protective effect, suggesting potential SKAT inflation. The nominally significant associations in ACAT-V and burden tests suggest these associations are robust, providing supporting evidence of true associations. Replication would be the gold standard to validate the association between *SMAD2* burden and IA, but no comparable cohort with whole exome or genome sequencing is currently available, making replication a goal for future research. Second, the unbalanced case/control ratio (1656 cases vs 391,948 controls) could increase type I error rates. We mitigated this by using robust Burden, SKAT, and ACAT-V tests as implemented in RVAT. Third, the generalizability of our findings is limited by the exclusive inclusion of individuals of European ancestry, which may not reflect non-European populations. Replication in other ancestries is needed to extrapolate the results. Fourth, since participants of the UK Biobank on average live a healthier lifestyle than the rest of the population, some caution is warranted in the interpretation of the results. While overall observations are generalizable to the European population, effect sizes may differ in individuals with less healthy lifestyles. Fifth, despite efforts to aggregate rare variants in burden tests, for some genes, only one or few variants passed selection criteria. This may have resulted in false negatives. Future studies with larger sample sizes or more informed variant selection may uncover additional associations missed in this study.

Our findings provide evidence that genetic variants in *PKD1* and *SMAD2* mechanistically underlie the risk of IA, thereby providing supporting evidence that screening for IA in patients with ADPKD and LDS may be beneficial. For ADPKD, previous studies have already shown that screening for IA can be cost-effective,^29,30^ whereas for LDS, screening is advised based on expert opinion.^31^ Furthermore, the finding that carriers of rare, damaging variants in *PKD1* and *SMAD2* were at increased risk of IA even in the absence of a diagnosis of ADPKD or LDS, suggests the potential for genetic screening in ASAH patients, since their family members may be at risk of IA/ASAH if they are carriers as well. Future research should assess the yield of such screening.

In conclusion, our results provide insight into the association between rare, damaging genetic variants in the genes *PKD1* and *SMAD2*, highlighting the shared etiology of IA with ADPKD and LDS, respectively. Moreover, we found that genes *PKD1* and *SMAD2* are associated with IA in patients without a diagnosis of ADPKD or LDS. Based on these findings, genetic testing for these variants may be considered in patients with an unruptured IA or ASAH. Our results further provide supporting evidence that screening for IA may be beneficial in patients with LDS and ADPKD and their relatives who carry one of these variants.

## Supplemental Material

sj-docx-1-wso-10.1177_17474930251334501 – Supplemental material for Rare genetic variants in PKD1 and SMAD2 are associated with intracranial aneurysms in the general population Supplemental material, sj-docx-1-wso-10.1177_17474930251334501 for Rare genetic variants in PKD1 and SMAD2 are associated with intracranial aneurysms in the general population by Bibi M Wolters, Mark K Bakker, Kristiina Rannikmäe, Paul J Hop and Ynte M Ruigrok in International Journal of Stroke

sj-docx-2-wso-10.1177_17474930251334501 – Supplemental material for Rare genetic variants in PKD1 and SMAD2 are associated with intracranial aneurysms in the general population Supplemental material, sj-docx-2-wso-10.1177_17474930251334501 for Rare genetic variants in PKD1 and SMAD2 are associated with intracranial aneurysms in the general population by Bibi M Wolters, Mark K Bakker, Kristiina Rannikmäe, Paul J Hop and Ynte M Ruigrok in International Journal of Stroke

sj-xlsx-3-wso-10.1177_17474930251334501 – Supplemental material for Rare genetic variants in PKD1 and SMAD2 are associated with intracranial aneurysms in the general population Supplemental material, sj-xlsx-3-wso-10.1177_17474930251334501 for Rare genetic variants in PKD1 and SMAD2 are associated with intracranial aneurysms in the general population by Bibi M Wolters, Mark K Bakker, Kristiina Rannikmäe, Paul J Hop and Ynte M Ruigrok in International Journal of Stroke
