# Supplementary material for: Rare genetic variants in PKD1 and SMAD2 are associated with intracranial aneurysms in the general population
Source: Int J Stroke. 2025 Apr 2;20(8):1011–20. doi: 10.1177/17474930251334501 (PMC12446701; doi:10.1177/17474930251334501)
Supplement: sj-docx-1-wso-10.1177_17474930251334501 – Supplemental material for Rare genetic variants in PKD1 and SMAD2 are associated with intracranial aneurysms in the general population [file sj-docx-1-wso-10.1177_17474930251334501.docx]

## Single-variant association analysis

To test is an observed association between gene burden and IA is indeed driven by multiple variants, and not the result of one or a very small number of genetic variants in that gene, we performed a single-variant association analysis. All variants passing variant annotation and selection were selected for this analysis. We used plink v2.0 to perform a Firth logistic regression using the same covariates and phenotypes used for burden analysis.^1^

## Additional details on the gene-based tests

The Burden test aggregates all variants within a gene and then assesses their overall association with a phenotype, assuming that all variants exert the same direction of effect. The SKAT test allows variants to have different directions and magnitudes of effect. The ACAT-V test aggregates association test scores of individual variants, combining their p-values on gene-level. The ACAT-V test performs an aggregated test on variants with a MAC below a threshold (here, set at 10), and analyses this together with single-variant statistics of variants with a MAC above the threshold. This method exhibits increased power compared to other tests when dealing with a small number of causal variants within a gene. Employing various tests can indicate whether the results are robust to slight changes in the statistical approach as well as test different assumptions about the genetic architecture. We employed the robust versions of the Burden and SKAT tests available in the rare variant analysis toolkit (RVAT) to account for the unbalanced case/control ratio in our dataset.^2,3^ Similarly, we utilized the Firth version of the ACAT-V test, which executes single variant tests using Firth regression instead of single variant score tests, as Firth regression has been demonstrated to control type I error well for both balanced and unbalanced studies.^4^

## Additional statistical analyses

In addition to the main burden analysis testing for association with IA (defined as both UIA and ASAH), we performed analyses including only cases with an ASAH diagnosis versus controls. The inclusion and exclusion criteria for this analysis can be found in Supplemental Table 1. To explore whether an observed association between genetic variants and IA was independent of monogenic disorders that exhibit an inherent predisposition for IA, we validated our findings excluding patients with ADPKD, Ehlers-Danlos syndrome, Marfan’s syndrome, or LDS. See Supplemental Table 1 for the precise description of the exclusion criteria and Table 1 for the demographics of the resulting cohort.

Since the probability to observe an effect between rare variant burden and IA/ASAH depends on the selection of variants to include, we performed sensitivity analyses based on allele frequency, and on predicted impact. We tested these variant selections on the genes *PKD1* and *SMAD2* that were identified to have an association with IA in the main variant selection categories. First, we included low-frequency variants (minor allele frequency < 5%) to determine whether these variants contribute to the risk of IA in genes where rare or ultrarare variants play a role. Second, we created an additional variant impact category based on predictions by AlphaMissense.^5^ We obtained variant effect predictions based on variant position and allele change from <https://zenodo.org/records/8208688>. We selected variants with a AlphaMissense annotation “likely pathogenic” to be included in this analysis.

# References

1. Chang CC, Chow CC, Tellier LC, et al. Second-generation PLINK: rising to the challenge of larger and richer datasets. *Gigascience* 2015; 4: 7.

2. Zhao Z, Bi W, Zhou W, et al. UK Biobank Whole-Exome Sequence Binary Phenome Analysis with Robust Region-Based Rare-Variant Test. *Am J Hum Genet* 2020; 106: 3–12.

3. Kenna K, Hop P. rvat: Rare variant analysis toolkit.

4. Wang X. Firth logistic regression for rare variant association tests. *Front Genet*; 5. Epub ahead of print 2014. DOI: 10.3389/FGENE.2014.00187.

5. Cheng J, Novati G, Pan J, et al. Accurate proteome-wide missense variant effect prediction with AlphaMissense. *Science (1979)*; 381. Epub ahead of print 22 September 2023. DOI: 10.1126/science.adg7492.

1. Cheng J, Novati G, Pan J, et al. Accurate proteome-wide missense variant effect prediction with AlphaMissense. Science 2023;381:eadg7492.
