# Supplementary material for: Rare genetic variants in PKD1 and SMAD2 are associated with intracranial aneurysms in the general population
Source: Int J Stroke. 2025 Apr 2;20(8):1011–20. doi: 10.1177/17474930251334501 (PMC12446701; doi:10.1177/17474930251334501)
Supplement: sj-docx-2-wso-10.1177_17474930251334501 – Supplemental material for Rare genetic variants in PKD1 and SMAD2 are associated with intracranial aneurysms in the general population [file sj-docx-2-wso-10.1177_17474930251334501.docx]

# Supplemental Tables

**Supplemental Table 1 - Selection and exclusion criteria for additional statistical analyses, including the positive control analysis with polycystic kidney disease as outcome, the analysis excluding patients diagnosed with disorders predisposing to intracranial aneurysms, and the analysis with aneurysmal subarachnoid hemorrhage as outcome.**

| **Analysis** | **Data-Fields** | **Case selection criteria** | **Exclusion criteria** |
| --- | --- | --- | --- |
| **Positive control** | ICD10 diagnosis – main (41202)  ICD10 diagnosis – secondary (41204)  ICD10 cause of death – underlying (40001)  ICD10 cause of death – contributory (40002) | **Q61.2** – polycystic kidney disease, adult type  **Q61.3** – polycystic kidney disease, unspecified |  |
|  | Genetic ethnic grouping (22006) |  | **not ‘Caucasian’** |
| **Excluding patients diagnosed with disorders predisposing to intracranial aneurysms** | ICD10 diagnosis – main (41202)  ICD10 diagnosis – secondary (41204)  ICD10 cause of death – underlying (40001)  ICD10 cause of death – contributory (40002) | **I671** - *cerebral aneurysm, unruptured*  **I60** - *subarachnoid hemorrhage* | **Q61.1** *polycystic kidney, infantile type*  **Q61.2**  *polycystic kidney, adult type*  **Q61.3**  *polycystic kidney, unspecified*  **Q79.6**  *Ehlers-Danlos syndrome*  **Q87.4**  *Marfan’s syndrome*  **Q87.8**  *other specified congenital malformation syndromes, not elsewhere classified; includes Loeys-Dietz syndrome* |
|  | Genetic ethnic grouping (22006) |  | **not ‘Caucasian’** |
| **ASAH as outcome** | Source of subarachnoid hemorrhage report (42013) | **‘hospital admission’**  **‘death only’**  **‘hospital primary’**  **‘death primary’**  **‘hospital secondary’**  **‘death contributory’** | **‘self-reported only’** |
|  | Genetic ethnic grouping (22006) |  | **not ‘Caucasian’** |

ASAH: aneurysmal subarachnoid hemorrhage; ICD: international classification of diseases. Individuals meeting one or more of the case selection criteria are classified as cases. Individuals meeting one or more of the exclusion criteria are excluded from the analysis.

**Supplemental Table 2 – Data description of burden analyses. Nvar: number of variants; caseCarriers: number of cases carrying one or more variants (caseCarriers); ctrlCarriers: number of controls carrying one or more variants.**

|  | **nvar** | **caseCarriers** | **ctrlCarriers** |
| --- | --- | --- | --- |
| ***ADAMTS15*** |  |  |  |
| **rare** |  |  |  |
| high | 21 | 0 | 55 |
| moderate+high | 554 | 72 | 14582 |
| **ultrarare** |  |  |  |
| high | 20 | 0 | 44 |
| moderate+high | 469 | 6 | 1306 |
| ***ANGPTL6*** |  |  |  |
| **rare** |  |  |  |
| high | 42 | 6 | 1199 |
| moderate+high | 374 | 31 | 9693 |
| **ultrarare** |  |  |  |
| high | 30 | 1 | 95 |
| moderate+high | 294 | 4 | 817 |
| ***ANK3*** |  |  |  |
| **rare** |  |  |  |
| high | 55 | 0 | 69 |
| moderate+high | 1841 | 177 | 46882 |
| **ultrarare** |  |  |  |
| high | 55 | 0 | 69 |
| moderate+high | 1557 | 19 | 3706 |
| ***ARHGEF17*** |  |  |  |
| **rare** |  |  |  |
| high | 39 | 0 | 61 |
| moderate+high | 1226 | 174 | 33876 |
| **ultrarare** |  |  |  |
| high | 39 | 0 | 61 |
| moderate+high | 999 | 8 | 2495 |
| ***COL3A1*** |  |  |  |
| **rare** |  |  |  |
| high | 26 | 1 | 30 |
| moderate+high | 605 | 97 | 20854 |
| **ultrarare** |  |  |  |
| high | 26 | 1 | 30 |
| moderate+high | 530 | 6 | 1154 |
| ***FBN1*** |  |  |  |
| **rare** |  |  |  |
| high | 17 | 1 | 55 |
| moderate+high | 1052 | 57 | 14498 |
| **ultrarare** |  |  |  |
| high | 16 | 1 | 24 |
| moderate+high | 893 | 12 | 2228 |
| ***FMNL2*** |  |  |  |
| **rare** |  |  |  |
| high | 32 | 1 | 84 |
| moderate+high | 453 | 94 | 22061 |
| **ultrarare** |  |  |  |
| high | 31 | 0 | 56 |
| moderate+high | 384 | 2 | 848 |
| ***LOXL2*** |  |  |  |
| **rare** |  |  |  |
| high | 44 | 2 | 440 |
| moderate+high | 472 | 30 | 6622 |
| **ultrarare** |  |  |  |
| high | 40 | 0 | 98 |
| moderate+high | 377 | 4 | 1035 |
| ***NFX1*** |  |  |  |
| **rare** |  |  |  |
| high | 38 | 1 | 84 |
| moderate+high | 526 | 36 | 9835 |
| **ultrarare** |  |  |  |
| high | 38 | 1 | 84 |
| moderate+high | 450 | 2 | 1123 |
| ***PCNT*** |  |  |  |
| **rare** |  |  |  |
| high | 161 | 3 | 767 |
| moderate+high | 2132 | 297 | 69562 |
| **ultrarare** |  |  |  |
| high | 145 | 2 | 299 |
| moderate+high | 1685 | 15 | 4269 |
| ***PKD1*** |  |  |  |
| **rare** |  |  |  |
| high | 60 | 1 | 150 |
| moderate+high | 3672 | 490 | 109600 |
| **ultrarare** |  |  |  |
| high | 56 | 1 | 74 |
| moderate+high | 2788 | 46 | 7427 |
| ***PKD2*** |  |  |  |
| **rare** |  |  |  |
| high | 18 | 1 | 38 |
| moderate+high | 502 | 56 | 16805 |
| **ultrarare** |  |  |  |
| high | 18 | 1 | 38 |
| moderate+high | 409 | 2 | 1083 |
| ***PMEPA1*** |  |  |  |
| **rare** |  |  |  |
| high | 10 | 0 | 17 |
| moderate+high | 152 | 19 | 4359 |
| **ultrarare** |  |  |  |
| high | 10 | 0 | 17 |
| moderate+high | 129 | 2 | 328 |
| ***PPIL4*** |  |  |  |
| **rare** |  |  |  |
| high | 45 | 3 | 340 |
| moderate+high | 232 | 12 | 2736 |
| **ultrarare** |  |  |  |
| high | 37 | 0 | 102 |
| moderate+high | 190 | 3 | 449 |
| ***RNF213*** |  |  |  |
| **rare** |  |  |  |
| high | 176 | 6 | 690 |
| moderate+high | 2799 | 324 | 73033 |
| **ultrarare** |  |  |  |
| high | 163 | 1 | 356 |
| moderate+high | 2257 | 19 | 5807 |
| ***SMAD2*** |  |  |  |
| **rare** |  |  |  |
| high | 9 | 1 | 11 |
| moderate+high | 127 | 5 | 558 |
| **ultrarare** |  |  |  |
| high | 9 | 1 | 11 |
| moderate+high | 122 | 4 | 221 |
| ***SMAD3*** |  |  |  |
| **rare** |  |  |  |
| high | 5 | 0 | 6 |
| moderate+high | 136 | 3 | 449 |
| **ultrarare** |  |  |  |
| high | 5 | 0 | 6 |
| moderate+high | 125 | 3 | 257 |
| ***TBC1D2*** |  |  |  |
| **rare** |  |  |  |
| high | 52 | 0 | 280 |
| moderate+high | 518 | 76 | 17113 |
| **ultrarare** |  |  |  |
| high | 46 | 0 | 113 |
| moderate+high | 390 | 2 | 1057 |
| ***TGFB2*** |  |  |  |
| **rare** |  |  |  |
| high | 6 | 0 | 7 |
| moderate+high | 169 | 53 | 9673 |
| **ultrarare** |  |  |  |
| high | 6 | 0 | 7 |
| moderate+high | 148 | 0 | 334 |
| ***TGFB3*** |  |  |  |
| **rare** |  |  |  |
| high | 11 | 0 | 14 |
| moderate+high | 170 | 3 | 1014 |
| **ultrarare** |  |  |  |
| high | 11 | 0 | 14 |
| moderate+high | 148 | 1 | 368 |
| ***TGFBR1*** |  |  |  |
| **rare** |  |  |  |
| high | 16 | 0 | 30 |
| moderate+high | 204 | 14 | 4919 |
| **ultrarare** |  |  |  |
| high | 16 | 0 | 30 |
| moderate+high | 175 | 2 | 352 |
| ***TGFBR2*** |  |  |  |
| **rare** |  |  |  |
| high | 12 | 0 | 23 |
| moderate+high | 242 | 25 | 6130 |
| **ultrarare** |  |  |  |
| high | 12 | 0 | 23 |
| moderate+high | 211 | 1 | 576 |
| ***THSD1*** |  |  |  |
| **rare** |  |  |  |
| high | 37 | 1 | 232 |
| moderate+high | 432 | 37 | 8889 |
| **ultrarare** |  |  |  |
| high | 33 | 1 | 80 |
| moderate+high | 361 | 3 | 919 |
